# Music and neuro-cognitive deficits in depression

**DOI:** 10.3389/fpsyg.2022.959169

**Published:** 2022-08-04

**Authors:** Prathima A. Raghavendra, Shantala Hegde, Mariamma Philip, Muralidharan Kesavan

**Affiliations:** ^1^Clinical Neuropsychology and Cognitive Neuroscience Centre, Music Cognition Laboratory, Department of Clinical Psychology, National Institute of Mental Health and Neuroscienes (NIMHANS), Bengaluru, India; ^2^Clinical Neuropsychology and Cognitive Neuroscience Centre, Department of Clinical Psychology, Music Cognition Laboratory, Wellcome Trust/DBT India Alliance CPH - Intermediate Fellow (IA/CPHI/17/1/503348), National Institute of Mental Health and Neuroscienes (NIMHANS), Bengaluru, India; ^3^Department of Biostatistics, NIMHANS, Bengaluru, India; ^4^Department of Psychiatry, NIMHANS, Bengaluru, India

**Keywords:** depression, neuro-cognition, music-cognition, attention, executive functions, memory

## Abstract

**Background:**

Cognitive deficits are one of the core features of major depressive disorder (MDD) that play crucial role in functional recovery. Studies have explored cognitive deficits in MDD, however, given inconsistent results, especially in mild-moderate MDD. Recently, studies have explored music as cognitive ability in various clinical conditions. In MDD, large focus has been on evaluating emotion deficits and just a handful on music cognition. With growing evidence on use of music based intervention to target cognitive deficits, it is imperative to explore nature of music cognitive ability in MDD.

**Aim:**

To examine musical and neuro-cognitive deficits in patients with mild-moderate MDD.

**Methods:**

Patients diagnosed with mild or moderate MDD (*n* = 19) and matched healthy controls (HC) (*n* = 18) were evaluated on selected tests from NIMHANS Neuropsychological test battery and Montreal battery for evaluation of amusia (MBEA).

**Results:**

MDD group performed significantly lower than HC on working memory (*p* = 0.007), verbal learning (*p* = 0.02) and retention (*p* = 0.03). Three indices were computed for a comprehensive evaluation. Groups did not differ significantly in any of the indices*-* focused attention, executive function, learning and memory as well as on music cognition. Focused attention and memory index predicted music cognition in HC and the combined group (MDD + HC) (*p* < 0.01). Attention alone contributed to 62.1% of variance in music cognition. Similarly, music cognition significantly predicted focused attention (*p* < 0.01).

**Conclusion:**

Individuals with mild-moderate MDD show significant deficits in working memory, verbal learning and memory, however, not in music cognition. There exists a significant relationship between music cognition and attention, which could be implicated in use of music interventions to ameliorate cognitive deficits. Limitations of study include small sample size and heterogeneity. Future studies on larger cohort examining musical emotion perception and neurocognition is imperative to have deeper understanding of this debilitating condition.

## Introduction

Cognitive dysfunction is one of the core features of major depressive disorder (MDD) and several studies have been carried out exploring the nature of neuro-cognitive deficits in MDD. Reviews and meta-analytic studies have reported significant deficits in cognitive domains such as attention, executive functions, verbal and visual retention of information in MDD (McDermott and Ebmeier, 2009; Rock et al., 2014; Kaser et al., 2017). Neuro-cognitive deficits impact functional recovery and quality of life in patients with depression (Jaeger et al., 2006; Conradi et al., 2011; Roca et al., 2015). These deficits are observed even in first episode depression and can persist during remission (Airaksinen et al., 2004; Biringer et al., 2007; Gonda et al., 2015). Interestingly, studies that have examined varying levels of depression severity have revealed contradictory findings owing to methodological differences (Landrø et al., 2001; Murphy et al., 2003; Shenal et al., 2003; Gorlyn et al., 2006; Russo et al., 2015). Some studies report that patients with mild depression perform equal to the controls on cognitive tests (Airaksinen et al., 2004). In comparison to other neuropsychiatric conditions such as schizophrenia, there are limited studies examining neurocognitive profile in patients with mild to moderate MDD and there seems to be no commonly agreed neurocognitive profile characterizing depression (Gorlyn et al., 2006).

In addition to neurocognitive functions, over the last one and a half decade, studies have examined emotion perception deficits in depression (Bouhuys et al., 1995; Kan et al., 2004; Péron et al., 2011; Van Vleet et al., 2019). In recent times, studies have been initiated to also examine music cognitive deficits and music emotion perception deficits in depression. The reasons for this are twofold. One, music cognitive deficits are being studied in par with other neurocognitive functions that have been traditionally evaluated. Two, music is a strong elicitor of emotion. Hence deficits in emotion perception and use of music-based intervention to help alleviate mood symptoms are being explored quite rigorously in the recent past (Naranjo et al., 2011; Aalbers et al., 2017). Yet, very little is known about music cognitive deficits in depression such as deficits in perception of musical features such as melodic contour, scale, rhythm perception and rhythmic contour.

### Music cognition and major depression

Music has always been an integral part of human society (Fitch, 2006) and in the recent past, investigation of music perception and processing has been used as a key to understand human brain. Music perception, like language processing, involves hierarchically organized network and activation of cortical and subcortical brain areas that govern auditory perception, semantic processing, attention, memory and emotion (Koelsch, 2010; Sarkamo et al., 2013). Processing of music is thus considered emotionally laden, neuro-cognitive process. In depression, so far studies have mainly focused on understanding deficits in musical emotion and not so much on music cognition (Gotlib et al., 2004; Punkanen et al., 2011).

Music cognition is currently a growing research area bringing an information processing approach, combining music theory with cognitive neuro-science in understanding music perception. Only in the recent times, studies have explored music cognitive deficits in depression similar to other neurological and neuropsychiatric conditions such as stroke, Parkinson’s disease, schizophrenia (Schuppert et al., 2000; Särkämö et al., 2009, 2010; Hatada et al., 2014; Wen et al., 2014; Biswas et al., 2016; Fujito et al., 2018). Deficits in musical expression such as disrupted tempo while playing instrument, deficits in identifying and discrimination of music elements such as pitch, rhythm and melody have been reported in patients with depression (Steinberg and Raith, 1985; Schwenzer et al., 2012). A longitudinal follow up study reported impairment in melody comparison, rhythm perception and emotional categorization of music, which improved with symptom reduction during follow up in patients with severe depression (Reker et al., 2014). Musical deficits may be implicated in depression in several ways. Studies have linked musical deficits to deficits in vocal emotion perception (Nussbaum and Schweinberger, 2021). Individuals with MDD are reported to have difficulties in processing emotions presented *via* facial, vocal and musical stimuli (Naranjo et al., 2011). These deficits can interfere with social functioning and their interpersonal relationships. In addition, music processing such as rhythm perception and entrainment are seen as very basic perceptual processes occurring even before conscious cognitive mechanisms. Ability to perceive beat and rhythm is said to optimize attentional resources and has its links to core cognitive mechanisms such as attention and information processing (Jones, 1976; Large and Jones, 1999; Bolger et al., 2013; Smith et al., 2014; Bharathi et al., 2019). It is also important to note that, music perception deficits can be disabling even in individuals who are not professional musicians. In other words, musical deficits are known to have significant implications in the non-musical domains of cognitive functioning and has an impact on the individuals’ overall functioning including emotional and social functioning.

Given these common theoretical links and neural substrates between music and cognition, it opens up clear possibility of dense association between them. It is also interesting to note that musical activities are proven to be efficacious in improving cognitive functions in various psychiatric and neurological conditions (Maratos et al., 2008; Bradt et al., 2010; Thaut, 2010; Hegde, 2014; Leubner and Hinterberger, 2017; Massaia et al., 2018). However, studies exploring the relationship between music and neuro-cognition systematically are scarce. Deeper understanding about this association would further provide evidence and expand possibilities of development of music-based interventions targeting neurocognitive deficits in patients with depression.

Given this background and lack of any systematic study from the Indian subcontinent, the current study was carried out with the aim of exploring music-cognitive and neuro-cognitive deficits in patients with mild-moderate MDD.

## Materials and methods

### Participants

Patients aged between 18 and 45 years with formal education of 07 years, diagnosed with mild or moderate MDD as per the 10th revision of International Classification of diseases (ICD-10) (*n* = 19) and age, sex and education matched healthy controls (HC) (*n* = 18) comprised the sample. Patients were recruited from the outpatient services of a tertiary mental health and neuroscience institute, the National Institute of Mental Health and Neuro Sciences. Clinical evaluation of all the patients and confirmation of the diagnosis was carried out by a qualified psychiatrist (MK). Patients diagnosed with depressive episode as part of bipolar disorder, and those with suicidal attempts or tendencies were excluded from the study. Age, education and sex matched HC were recruited from the community. Individuals with any past history of other neurological/psychiatric illness or medical comorbidities were excluded from the study. All the participants were right-handed as screened on Edinburgh Handedness Inventory (Oldfield, 1971) and had no formal training in music or any other performing art forms for more than 2-years.

### Procedure

The study protocol was approved by the Institute’s Ethics Committee. Written informed consent was obtained from all participants. The socio demographic and clinical details of the patient group were recorded. MDD and HC group were assessed on the below mentioned tools over 1–2 sessions, with adequate rest period in between. The assessments were carried out in a span of two session on a few patients who reported fatigue. Patients were debriefed about the findings and feedback was given after the assessment were completed.

### Tools/scales

A socio-demographic sheet, a clinical data sheet and music behavior data sheets were prepared by the researcher which included basic demographic details, clinical details and musical preferences of the participants. A music behavior data sheet was prepared to collect information on participants’ musical preferences, details related to training, hours of listening, genre of preference and their perception and attitudes toward using music as a treatment modality. Participants were also asked to list subjectively perceived cognitive deficits and rate them on scale of 1–10, greater score corresponding to higher deficits.

The Hamilton Depression Rating Scale (HAMD) (Hamilton, 1986) was used to assess symptom severity of depression in patients.

Montreal Battery for Evaluation of Amusia (MBEA) was used to measure the music cognitive abilities. The MBEA allows the diagnosis amusia by assessing musical abilities related to six components of musical processing presented in the neuropsychological model of musical cognitive processing, namely: Contour, Scale, Interval, Rhythm, Meter, and Musical Memory (Peretz et al., 2003).

Selected tests from NIMHANS Neuropsychological test battery and Wechsler’s Memory Scale (Pushpalatha, 2004; Rao et al., 2004) were used to evaluate the neurocognitive functions. The tests used to assess the neurocognitive functions were as follows—Color trails test (D’Elia et al., 1996) to assess focused attention and cognitive flexibility (Color trails-2), Verbal N back test (Smith and Jonides, 1999) to assess verbal working memory, Spatial Span test, WMS-III India (Pushpalatha, 2004) to assess visual working memory, Stroop color-word test (Golden and Freshwater, 2002) to assess response inhibition, Rey’s Auditory Verbal Learning test (RAVLT) (Maj et al., 1994) to assess verbal learning and memory, and Rey’s Osterrieth Complex Figure Test (CFT) (Rey, 1941) to assess visual construction and memory.

### Statistical analysis

Data was coded for analysis using SPSS version 22.0 for Windows. Descriptive statistics such as mean, standard deviation and percentages were used to describe the socio-demographic data. Normality of data was assessed using Shapiro Wilks Test and Kolmogorov Smirnov Test.

#### Computation of neurocognitive indices

Six tests were used to measure various domains of neurocognitive functions. In order to compare the groups on neurocognitive domains—focused attention, executive functions and memory, three composite neurocognitive indices were obtained. The raw scores of each of the test were rescaled using min-max normalization to make the scores of different tests comparable. Mix-max normalization is a normalization strategy that scales the data between 0 and 1. It was calculated by subtracting the raw score from minimum value of the data set and then dividing it by the range (maximum value-minimum value). Composite executive function score was obtained by averaging the normalized scores of color trails 2, spatial span test, verbal N-Back 2 (hit score) and Stroop test. Composite learning and memory score were obtained by averaging normalized scores of AVLT (AVLT total, AVLT immediate recall and AVLT delayed recall) and CFT (CFT copy, CFT immediate recall and CFT delayed recall scores). Normalized score of Color trails 1 was considered as the focused attention score.

The performance of MDD and HC groups were compared on individual neurocognitive domains, neurocognitive indices and music cognition. Independent sample *t*-test was used to compare the groups for variables that assumed normality and Mann-Whitney *U*-test was applied to variables that violated the assumptions of normality. Welch Correction was applied wherever homogeneity of variance was not met. Appropriate correlation tests were used to understand the relationship between neurocognitive variables and music cognition. A stepwise linear regression was attempted in order to study the relationship between neurocognitive indices and music cognition.

## Results

### Socio-demographic and clinical status of the participants

Sample comprised of 19 patients diagnosed with MDD [MDD: *n* = 19; M: *F* = 7:12; Mean age (years) 28 ± 6] and age, sex and education matched healthy participants [HC: *n* = 18; M: *F* = 7:11 Mean age (years) 28.1 ± 7.5]. The two groups did not differ significantly on any of the socio-demographic variables (Table 1). Table 2 shows clinical status of MDD group. Patient sample consisted of 6 (31.57%) patients with mild depression and 13 patients (68.42%) with moderate depression. The mean age of onset was 24.50 ± 7.15 years with a range of 13–43 years. Eleven patients (57.9%) were receiving individual psychotherapy. The mean HAM-D score was 14.3 ± 2.06, with range of 10–17. The mean score of subjectively reported cognitive deficits was found to be 6.10 ± 1.60. Patients reported difficulties mostly in domain of concentration and memory (especially remembering conversations, instructions).

**TABLE 1:** Socio-demographic details.

Variables	Sub-categories		Group		t/χ^2^
			MDD (*n* = 19)	HC (*n* = 18)	
Age (in years)		Mean (S.D.)	28.0 (6.0)	28.16 (7.57)	0.074
Gender	Male	Frequency	7	7	0.016
		%	36.8	38.19	
	Female	Frequency	12	11	
		%	63.2	61.1	
Marital status	Married	Frequency	8	6	0.302
		%	42.1	33.3	
	Unmarried	Frequency	11	12	
		%	57.9	66.7	
Education (in years)		Mean (SD)	17.0 (2.13)	17.56 (2.13)	0.761
Employment	Unemployed	Frequency	10	8	0.248
		%	52.6	44.4	
	Employed	Frequency	9	10	
		%	47.4	55.6	

**TABLE 2:** Clinical status of the MDD group (*n* = 19).

Variable	Response categories	Descriptive	Value
Depression—severity	Mild	Frequency	06
		%	31.57
	Moderate	Frequency	13
		%	68.42
Duration	No. of years	Mean (SD)	3.51 (4.6)
Age of onset	In years	Mean (SD)	24.5 (7.15)
Scores on HAM-D	HAM-D total	Mean (SD)	14.2 (2.06)
Subjective cognitive complaints (on scale of 1–10; ten being severe deficits)		Mean (SD)	6.10 (1.6)

HAM-D, Hamilton Depression Rating Scale.

### Musical background and preferences of participants

As noted using the music behavior data sheet, a large percentage of both MDD (94.8%) and HC (100%) reported that they enjoyed listening to music. There was no significant difference between the two groups in terms of hours spent listening to music. Most participants (52.6% MDD vs. 50% HC) listened to music for more than 3 h in a week. Forty two percent of MDD patients and 33% of HC group had family members who were trained in music. Majority of the MDD and HC participants preferred concentrating on both lyrics and tune while listening to music (84% MDD vs. 89% HC). Also, majority of the participants opined that music can be used in treatment of psychiatric disorders. Participants self-reported a subjective evaluation of their ability to follow a rhythm, and keep a tune on a scale of 1–5, greater score corresponding to greater ability. There were no significant differences between MDD and HC on self-reported ability to remember tunes (3.5 HC vs. 3.8 MDD) as well as to keep rhythm (3.9 HC vs. 2.9 MDD).

### Neurocognitive profile of major depressive disorder and healthy controls group

Tables 3, 4 shows the comparative performance of MDD and HC on tests of neurocognitive functioning. MDD group did not significantly differ from HC group on focused attention, executive function and learning and memory indices in general though MDD group obtained lower index scores as compared to HC. However, when individual test performance was compared between MDD and HC group, it was found that, the MDD group performed significantly lower on tasks assessing working memory (*p* = 0.007; < 0.01 level), verbal learning (*p* = 0.02; at 0.05 level) and delayed recall (AVLT) (*p* = 0.035; 0.05 level).

**TABLE 3:** Comparison between MDD and HC group on various neurocognitive tests.

Variable	Mean (SD)	Group		*T*-value	*p*-Value
		MDD (*n* = 19)	HC (*n* = 18)		
Color trails I	Mean (SD)	60.26 (13.76)	56 (13.95)	0.936	0.356
Stroop test (Interference)	Mean (SD)	0.74 (6.3)	–1.43 (3.84)	–1.25	0.21
Complex figure test IR	Mean (SD)	23.84 (4.96)	24.22 (3.7)	–0.263	0.79
Complex figure test DR	Mean (SD)	23.03 (5.03)	23.89 (3.55)	–0.60	0.55

**Variable**	**Md (Q1,Q3)**	**MDD (*n* = 19)**	**HC (*n* = 18)**	***U*-value**	***p*-Value**

Color trails II	Md (Q1–Q3)	94 (80–102)	96.5 (76.5–102.7)	167	0.903
N- Back I hits	Md (Q1–Q3)	9 (8–9)	9 (9–9)	136.0	0.136
N-Back 2 hits	Md (Q1–Q3)	7 (7–8)	7 (6.75–8)	167.0	0.896
N-Back 2 errors	Md (Q1–Q3)	3 (2–3)	3 (1.75–4)	170.5	0.988
Spatial span	Md (Q1–Q3)	16 (14–18)	19 (16.75–21)	83.5	0.007*
AVLT total	Md (Q1–Q3)	294 (62–322)	331 (217–354.25)	94.50	0.02*
AVLT DR	Md (Q1–Q3)	13 (11–15)	15 (14–15)	105.5	0.035*
AVLT LTPR	Md (Q1–Q3)	100 (84.6–100)	100 (100–100)	122.0	0.065
Complex figure test copy	Md (Q1–Q3)	36 (34–36)	36 (35–36)	136.5	0.217

IR, Immediate recall; DR, Delayed Recall; AVLT, Auditory Verbal learning test. *p < 0.05.

**TABLE 4:** Comparison between MDD and HC group on neurocognitive indices.

Variable	Mean (SD)	Group		*T*-Value	*p*-Value
		MDD (*n* = 19)	HC (*n* = 18)		
Focused attention	Mean (SD)	0.52 (0.17)	0.47 (0.17)	0.936	0.356

**Variable**	**Md (Q1,Q3)**	**MDD (*n* = 19)**	**HC (*n* = 18)**	***U*-value**	***p*-Value**

Executive function index	Md (Q1–Q3)	0.48 (0.06–0.65)	0.50 (0.26–0.68)	150	0.523
Memory index	Md (Q1–Q3)	0.66 (0.20–0.88)	0.69 (0.38–0.81)	141	0.361

### Music cognition among major depressive disorder and healthy controls group

Table 5 shows the comparative performance of MDD and HC on test of music cognition. The groups did not significantly differ in their abilities to perceive musical differences on any of the music cognition domains– scale, contour, interval, rhythm, meter and memory. However, on mean comparison it is found that, the MDD group had underperformed (had lower scores) on all domains of music cognition.

**TABLE 5:** Comparison between MDD and HC groups on montreal battery of Amusia.

Variable	Mean (SD)	Group		*t*-Value	*p*-Value
		MDD (*n* = 19)	HC (*n* = 18)		
Scale	Mean (SD)	21.68 (5.18)	22.22 (5.28)	–0.313	0.756
Contour	Mean (SD)	22.26 (3.98)	23.06 (4.93)	–0.539	0.593
Interval	Mean (SD)	21.37 (3.98)	22.33 (3.94)	–0.741	0.464
Rhythm	Mean (SD)	24.95 (4.65)	26.06 (3.46)	–0.826#	0.415
Meter	Mean (SD)	19.89 (3.7)	21.61 (4.54)	–1.264	0.214
Memory	Mean (SD)	23.47 (3.13)	23.78 (3.66)	–2.272	0.787
Total Score	Mean (SD)	133.63 (18.4)	139.06 (1,952)	–0.870	0.390

# Welch correction for non-homogeneity of variance.

### Relationship between neuro-cognition and music cognition

Stepwise linear regression was carried out to explore relationship between music-cognition and neurocognition in combined (MDD + HC) groups and healthy control alone group (HC) (Table 6). Dependent variable to be entered in regression analysis was identified based on significant findings on the correlation analysis (Table 7). Focused attention and memory index were found to be significant predictors of music cognition in HC and the combined group (MDD + HC) (*p* < 0.01). Attention and memory indices together were seen to predict 60.1 and 77.1% of music cognition variance in combined group and HC group, respectively. Attention alone contributed to 62.1% of variance in music cognition among HC group. In addition, music cognition significantly predicted focused attention (*p* < 0.01) (Table 6).

**TABLE 6:** Stepwise linear regression between music cognition and neuro-cognition.

Significant predictor variable	Dependent variable	B		Std error		Adjusted R square		F (df)	
		
		Both groups	HC alone	Both groups	HC alone	Both groups	HC alone	Both groups	HC alone
Focused attention	Music cognition (MBEA total)	0.493	0.621	16.6	15.7	0.22	0.34	11.26**	10.02**
Focused attention and learning + memory index	Music cognition	0.60	0.771	15.5	13.2	0.32	0.54	9.62**	10.9**
Music cognition (MBEA total)	Focused attention	0.493	0.621	16.6	15.7	0.22	0.34	11.26**	10.02**

MBEA, Montreal Battery for evaluation of amusia total ; CT Color trails test **p<0.01.

**TABLE 7:** Spearman’s rho between Neurocognitive indices and music cognition.

Neurocognitive indices			Music cognition (total MBEA)
Attention index	HC	*r*	0.626**
		*p*	0.005
	MDD + HC	*r*	0.508**
		*p*	0.001
Executive function index	HC	*r*	0.319
		*p*	0.19
	MDD + HC	*r*	0.326*
		*p*	0.49
Learning and memory index	HC	*r*	0.32
		*p*	0.18
	MDD + HC	*r*	0.39**
		*p*	0.15

*p < 0.05 ; **p < 0.01.

## Discussion

The present study investigated music cognition and neuro-cognition in MDD compared to age, education and sex matched HC. It was observed that MDD underperformed on all the neurocognitive indices. The findings revealed significant differences between the groups on working memory, verbal encoding and retention. These findings are partly consistent with research findings reported in the field that have demonstrated presence of neurocognitive deficits in domains of executive functions (Harvey et al., 2005), attention and concentration (Kampf-Sherf et al., 2004), verbal and visual memory (Fossati et al., 1999; Zubieta et al., 2001). The findings from the present study did not show global cognitive deficits as documented in some of the previous studies. The circumscribed cognitive deficits could be explained by inconsistencies in association between depression and neuro-cognition in general (Austin et al., 2001; Wang et al., 2006; Gualtieri and Morgan, 2008). Most studies that have explored neurocognitive functioning in depression have not controlled for the severity of depression, which is known to have positive association with cognitive deficits (McDermott and Ebmeier, 2009). Patients with mild depression show lesser degree of deficits in executive function (Elderkin-Thompson et al., 2004).

Findings from the present study showed no significant differences between HC and MDD in the domain of music cognition. Very few studies are available that have explored music as cognitive variable and they have established music cognition deficits and evidences of abnormalities in auditory processing in certain conditions including depression (Steinberg and Raith, 1985; Michael et al., 2004; Tollkötter et al., 2006; Chase et al., 2010; Reker et al., 2014; Kraus et al., 2019). Music perception ability has been closely linked with neurocognitive functions such as attention, executive functions and visuo-spatial abilities (Särkämö et al., 2009; Wen et al., 2014). The pitch, rhythm, contour and interval discrimination subtests along with memory subtest, places high demand on cognitive functions including attention and working memory. In the current study, the MDD group did not have global cognitive impairment and seems to have relatively preserved cognitive abilities. This could one of the possible reasons for preserved musical abilities.

In this study we also observed a close link between focused attention with music cognition interestingly in combined group (MDD + HC) and HC alone, but not in MDD group. This could be explained by neurocognitive deficits in MDD group. Though, MDD and HC did not significantly differ on indices, individual domain analysis revealed deficits specifically in working memory, learning and memory.

In the current study, of all neurocognitive indices, focused attention was found to significantly predict music cognition. In addition, music cognition significantly predicted focused attention. Studies have shown bi-directional relationship between cognitive functions and music perception. Components of music such as timing, grouping and organization facilitates attentional processes by interacting with attention oscillators (Thaut, 2010; Thaut and Hoemberg, 2014). As mentioned earlier, ability to perceive rhythm could act as precursor to conscious cognitive processes including information processing, attention and memory. On other hand, It is also established that focusing and keeping track of music in time activates attentional and working memory network bilaterally, however, with activity in right hemisphere being more dominant (Sihvonen et al., 2017). Engaging in musical activities ranging from simple pitch and rhythm discriminating tasks to complex activities such as singing, listening, playing instrument, music production and improvisation can involve higher order cognitive processes.

In recent past, music-based interventions have been proven to be efficacious in improving neuro-cognitive functions including attention, executive functions and memory in several clinical conditions (Knott, 2017; Tumuluri et al., 2017; He et al., 2018; Jones et al., 2021). Cognitive remediation in depression in gaining its due importance in the field and music based interventions can prove to be beneficial. Although few in number, a few studies have shown positive results in depression as well in targeting cognition and emotional disturbances (Chu et al., 2014; Tai et al., 2015). Probably, a relatively well-preserved music cognitive ability can act as a mediating factor in effectiveness of music-based interventions in depression.

## Conclusion

In conclusion, patients with mild-moderate depression perform poorly than HC in neurocognitive domains such as working memory, verbal encoding and retention, however, do not significantly differ from HC in music cognitive ability. Neuro-cognition and music cognition are found to significantly relate to each other.

Current study can be considered as an exploratory study. The present study has certain limitations. The sample size was small leading to limited generalizability of the findings. The sample was heterogeneous in terms of certain clinical variables such as age of onset, duration of illness and medications/therapy. There was a preponderance of females over males in the study.

Most studies available have explored music emotion perception and processing. And, there is clear dearth of studies aiming at understanding music cognition deficits in relation to neuro-cognition in affective disorders. Further, studies can also explore music emotion processing and its links with music cognition and neuro-cognition in affective disorders. Most patients in the present study enjoyed listening to music and 90% of them endorsed the view that music could be a tool for psychological interventions in the disorder which also strengthens the need to explore further and integrate music-based interventions to routine treatment options, especially those rooted in neuroscientific evidence.

## Data availability statement

The raw data supporting the conclusions of this article will be made available by the authors, without undue reservation.

## Ethics statement

The studies involving human participants were reviewed and approved by the Institute Ethics Committee, National Institute of Mental Health and Neurosciences. The patients/participants provided their written informed consent to participate in this study.

## Author contributions

PAR worked on the research protocol, reviewed the literature, collected the data, carried out the data analysis and wrote the manuscript. SH conceptualized the study, designed the method, edited, and finalized the manuscript. MP contributed to study design, data analysis, and interpretation. MK contributed to the study design, clinical evaluation of patients recruited in the study, and contributed in finalizing the manuscript. The final article has been approved by all the authors of the current study.
